# Cardiac Arrhythmias In Congenital Heart Diseases

**Published:** 2009-11-01

**Authors:** Paul Khairy, Seshadri Balaji

**Affiliations:** 1 Adult Congenital Heart Center and Electrophysiology Service, Montreal Heart Institute, University of Montreal, Montreal, QC, Canada; 2 Division of Pediatric Cardiology, Department of Pediatrics, Oregon Health & Science University, Portland, OR, USA

**Keywords:** Congenital heart disease, Atrial arrhythmias, Ventricular arrhythmias, Sudden cardiac death

## Abstract

Arrhythmias figure prominently among the complications encountered in the varied and diverse population of patients with congenital heart disease, and are the leading cause of morbidity and mortality. The incidence generally increases as the patient ages, with multifactorial predisposing features that may include congenitally malformed or displaced conduction systems, altered hemodynamics, mechanical or hypoxic stress, and residual or postoperative sequelae. The safe and effective management of arrhythmias in congenital heart disease requires a thorough appreciation for conduction system variants, arrhythmia mechanisms, underlying anatomy, and associated physiology. We, therefore, begin this review by presenting the scope of the problem, outlining therapeutic options, and summarizing congenital heart disease-related conduction system anomalies associated with disorders of the sinus node and AV conduction system. Arrhythmias encountered in common forms of congenital heart disease are subsequently discussed. In so doing, we touch upon issues related to risk stratification for sudden death, implantable cardiac devices, catheter ablation, and adjuvant surgical therapy.

## Scope Of The Problem

Congenital heart disease is the most common form of birth defect, with an estimated 1-2% of live newborns afflicted by moderate or severe types   [1]. Arrhythmias figure prominently among the healthcare issues encountered and pose unique and diverse challenges [2,3]. The incidence of arrhythmias generally increases as the patient with congenital heart disease ages. Indeed, by adulthood, arrhythmias are the leading cause of morbidity and hospital admissions [4,5], and sudden death of presumed arrhythmic etiology is the most common cause of mortality  [6,7].

Arrhythmias may reflect congenitally malformed or displaced conduction systems, altered hemodynamics, mechanical and/or hypoxic stress, and/or residual or postoperative sequelae [5,8].  The entire gamut of arrhythmia subtypes may occur in patients with congenital heart disease, with several forms often coexisting. Bradyarrhythmias may involve disorders of the sinus node, atrioventricular (AV) node, His-Purkinje system, or intra-atrial propagation.  Junctional tachyarrhythmias are common, particularly in young post-operative patients [9]. Atrial tachyarrhymias are highly prevalent and may be mediated by accessory pathways, dual AV nodal reentry, twin AV nodes, macroreentrant circuits, automatic rhythms, or non-automatic foci. Atrial fibrillation is increasingly prevalent in the growing and aging population of adults with congenital heart disease. Ventricular arrhythmias are thought to be the leading cause of sudden death in several subtypes of congenital heart disease.

## Therapeutic options

Importantly, arrhythmias may herald a changing hemodynamic profile and should generally prompt a detailed work-up. The care of the patient with congenital heart disease and arrhythmias may involve pharmacological therapy, catheter ablation, implantable cardiac devices, and surgical interventions.

In the absence of specific evidence-based recommendations, pharmacological therapy is often guided by principles established in other forms of heart disease [10,11]. These include considerations regarding systemic ventricular dysfunction, sinus node disease, impaired AV node conduction, negative inotropic effects, and proarrhythmia. The comparative efficacy of antiarrhythmic agents remains poorly studied, with little data regarding dosing and toxicity for the various age groups with congenital heart disease. Amiodarone-associated thyroid dysfunction is common in adults with congenital heart disease, especially in women and those with complex cyanotic heart disease or univentricular hearts with Fontan palliation [12]. There is much interest in the new generation of class III antiarrhythmic agents that purport fewer multisystemic side-effects without increased mortality in the setting of left ventricular dysfunction. In a multicenter case series, dofetilide appeared to be a viable adjunct to catheter-based ablation and alternative pharmacological approaches for atrial arrhythmias in adult patients with congenital heart disease [10].

Anatomical complexities and vascular access issues may complicate catheter-based interventions and implantation of pacemakers or implantable cardioverter-defibrillators (ICD) [2,13,14]. Challenges in device therapy include circumventing obstructed vessels, conduits, or baffles; minimizing thromboembolic risk in the presence of intracardiac shunts; identifying appropriate candidates for cardiac resynchronization therapy and primary prevention ICDs; configuring adequate vectors for defibrillation despite small sizes and/or limited vascular access; and a high rate of inappropriate shocks and lead complications [2,13,15,16].  Although the success rate is modest, some patients may benefit from pacemakers with automated overdrive pacing algorithms to terminate atrial tachyarrhythmias [17].

With the advent of three-dimensional electroanatomic mapping and advances in catheter technology permitting larger and deeper lesions, transcatheter ablation has emerged as a promising alternative for many patients with tachyarrhythmias.  While acute success rates in dedicated centers are high, recurrences and the onset of new arrhythmias remain problematic, particularly in patients with Fontan palliation.  In certain circumstances, arrhythmia surgery, usually performed in conjunction with cardiac surgery for other indications, may complement less invasive options.

## Conduction System Considerations

### Sinus node

Most patients with congenital heart disease have a normally positioned sinus node. Exceptions include left juxtaposition of the atrial appendages, situs inversus, and heterotaxy syndromes. In left juxtaposition of the atrial appendages, both appendages are on the left side of the arterial pedicle [18]. The sinus node is displaced anteriorly and inferiorly, below the crista terminalis [19]. In atrial situs inversus, the atria are positioned in a mirror-image fashion, with a left-sided sinus node. Heterotaxy syndromes may generally be categorized as either right (asplenia syndrome) or left (polysplenia syndrome) atrial isomerism. Patients with right atrial isomerism often have bilateral sinus nodes. The governing node may shift from one to the other [20]. In left atrial isomerism, sinus nodes are either absent or hypoplastic and displaced posteroinferiorly  [21]. Congenital sinus node dysfunction is common  [21].

### AV node and His-Purkinje system

The AV conduction system may be displaced if atrial and ventricular septae are malaligned, AV arrangements are discordant, or if the heart is univentricular. As a general rule of thumb, if the AV conduction system is displaced, it also tends to be more fragile and susceptible to degeneration, placing patients at greater risk for AV block.

In atrioventricular canal defects (AVCD), the AV node is displaced inferiorly and posteriorly  [22]. The His-bundle extends along the lower rim of the ventricular septum. This inferior course and hypoplastic left anterior hemifascicle gives rise to the characteristic superior QRS axis. In congenitally corrected transposition of the great arteries, or L-TGA, the AV node is displaced anteriorly and laterally  [23]. An elongated and fragile His-bundle courses across the anterior rim of the pulmonary valve. If a ventricular septal defect is present, it continues along its superior border  [23]. In patients with tricuspid atresia, the AV node is typically found on the floor of the right atrium near a small dimple lined with endocardium  [24]. The course of the His-Purkinje system may vary but is typically further leftward and away from more anterior ventricular septal defects  [24]. For other forms of univentricular hearts, key determinants of the course of the AV conduction system include the direction of ventricular looping and morphology of the dominant ventricle  [25]. The AV conduction system is often displaced with AV discordance and AVCD. In L-looped single left ventricles, two AV nodes may be present  [26,27]. The elongated His-bundle may be susceptible to damage, with complete AV block  [26]. With ventricular D-looping and a dominant right ventricle, the AV node remains within Koch's triangle  [27].

## Arrhythmias In Specific Congenital Heart Disease Lesions

### Atrial septal defect (ASD)

Macroreentrant atrial circuits are the most frequent arrhythmias encountered in patients with secundum and sinus venosus ASDs. In the absence of surgical repair, typical cavo-tricupsid isthmus-dependent atrial flutter is the most common form. In the presence of atriotomy incisions, sutures, and/or patches, non-isthmus dependent macroreentrant circuits may occur or coexist with typical flutter. Common substrates include macroreentry along the lateral right atrial wall and double-loop or figure-of-eight circuits  [28,29].

The incidence of atrial arrhythmias increases with age and has been reported in 20% of adults  [30,31]. Although surgical closure may decrease atrial arrhythmias, it is less effective in older patients   [30-32]. In 218 adults with isolated ASDs, sustained atrial arrhythmias occurred in 19% prior to surgery: atrial flutter alone in 5%, atrial flutter and fibrillation in 2.8%, and atrial fibrillation alone in 11%  [30]. Over a post-surgical follow-up of 3.8 years, atrial arrhythmias persisted or recurred in 60% of patients diagnosed preoperatively, and 2.3% developed new-onset arrhythmias. All patients with post-surgical atrial arrhythmias were over 40 years of age at time of repair. In a subsequent study that randomized 521 adults over 40 years of age with a secundum or sinus venosus ASD to surgical closure versus medical therapy, no difference in atrial tachyarrhythmias were noted at a median of 7 years post-operatively  [31]. The impact of transcatheter ASD closure on atrial arrhythmias is less clear. In one series, all patients with persistent arrhythmias remained in atrial fibrillation or flutter after closure  [32].

### Ventricular septal defect (VSD)

In patients with unoperated VSDs, isolated premature ventricular contractions (PVC), couplets, and multiform PVCs are prevalent  [33]. Non-sustained or sustained ventricular tachycardia has been observed in 6% [33]. A higher mean pulmonary artery pressure is associated with high-grade ectopy [33]. Nevertheless, in the absence of Eisenmenger syndrome, sudden cardiac death is uncommon [34,35]. Late sudden death has been reported in 4% of patients following surgical repair [36,37]. Of 296 patients with surgical VSD closure between 1954 and 1960, 20% of patients had transpired by 30 years of follow-up [37]. Risk factors for mortality included surgical repair after 5 years of age, pulmonary vascular resistance greater than 7 Woods units, and complete heart block. In patients who undergo transcatheter VSD closure, AV block is a major concern, with an estimated incidence of 3-4% [38].

### Atrioventricular canal defect (AVCD)

In patients with AV canal defects, frequent ventricular ectopy has been described in 30%, while complex ventricular arrhythmias are predominantly confined to those with left ventricular dysfunction [39]. Persistent complete AV block occurs in 1% to 7% in the immediate post-operative period and approximately 2% thereafter [39,40]. Prolonged infra-Hisian conduction time may be a marker for increased risk of late AV block, even if the PR interval is normal [41]. In 18 patients with AVCDs, preoperative electrophysiologic studies revealed sinus node dysfunction in 1, supra-Hisian first degree AV block in 5, and intraatrial conduction delay in the majority [36]. Atrial fibrillation or flutter has been noted in 5% of patients after surgical repair [39,42].

**Figure 1:**
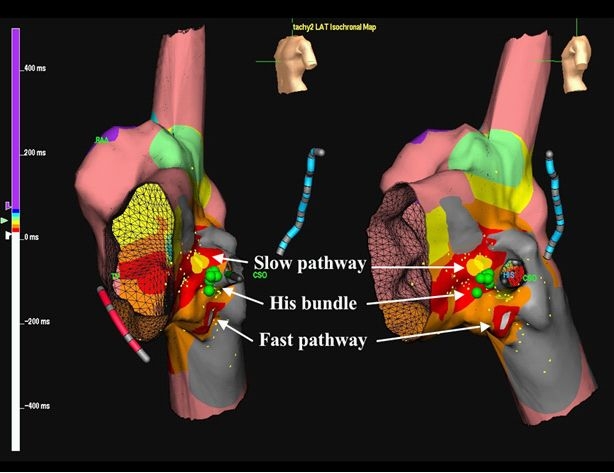
Cryomapping and cryoablation combined with 3D electroanatomic mapping in a patient with a partial AVCD. Three-dimensional electroanatomic maps of retrograde atrial activation during AV nodal reentrant tachycardia in left anterior oblique (Panel A) and left lateral (Panel B) views.  The blue decapolar catheter is positioned in the coronary sinus and red quadripolar catheter in the right ventricle.  Green circles indicate sites where His-bundle electrograms were recorded.  Local activation times are color-coded, with the site of earliest atrial activation in white, inferior to the infero-posteriorly displaced His-bundle.  The yellow circles represent the site of successful cryomapping and cryoablation of the slow pathway, superior to the His bundle.  RAA denotes right atrial appendage; CSO, coronary sinus ostium. Reproduced from Khairy P. et al. Partial atrioventricular canal defect with inverted atrioventricular nodal input into an inferiorly displaced atrioventricular node. Heart Rhythm 2007;4(3):355-8.43. Copyright (2007), with  permission from Elsevier.

Importantly, since the AV node is displaced just anterior to the mouth of the coronary sinus, interventional electrophysiologists should be cautious when ablating in the right inferior paraseptal region. For patients with cavo-tricuspid isthmus-dependent macroreentry, lateral ablation lines are generally preferred to prevent AV block. With dual AV node physiology, the slow pathway has been located superior to the His bundle, with the fast pathway inferior to the displaced AV node, as shown in Figure 1 [43].

### Left ventricular outflow tract obstruction

In patients with left ventricular outflow tract obstruction, ventricular arrhythmias have been related to severity of obstruction, increased wall stress, and left ventricular hypertrophy [5,44-46]. In a series of adults with unoperated aortic stenosis, 34% had high grade ventricular ectopy on Holter monitoring, compared to 6% of controls [44]. Correlations with lower left ventricular ejection fraction and higher wall stress were later demonstrated  [45,46]. Unfortunately, the risk of sudden death appears to persist despite surgical repair, warranting lifelong monitoring. Indeed, in a population-based study of sudden death after surgery for congenital heart disease, patients with aortic stenosis constituted the highest risk subgroup, with an incidence of 3% at 10 years and 20% at 30 years [6]. Aortic coarctation was also among the high risk lesions [6].

### Congenitally corrected transposition of the great arteries (L-TGA)

Patients with L-TGA have notoriously fragile AV conduction systems.  In 107 patients with L-TGA and mean age of 22 years, complete AV block occurred in 22% [47]. Risk of AV block was estimated to be 2% per year, irrespective of associated anomalies. Electrophysiological studies suggest that AV block occurs above or within the His bundle [48,49]. These studies are consistent with clinical and pathological observations. A stable narrow QRS escape rhythm often accompanies complete AV block [47] and fibrosis of the His bundle is observed histologically [49,50]. Since the AV node and His bundle are highly sensitive to catheter or surgical trauma, manipulation near these areas should be exercised with caution. Altered hemodynamics, such as the volume loading conditions of pregnancy, may place patients at higher risk of developing AV block. Complete AV block follows surgical repair of an associated VSD in over 25% [47,51].

### Ebstein's anomaly

In Ebstein's anomaly, the atrialized portion of the right ventricle is morphologically and electrically right ventricle but functionally right atrium [52]. Mechanical stimulation of the atrialized right ventricle may induce ventricular arrhythmias. Otherwise, spontaneous ventricular tachycardia is uncommon in the absence of associated malformations, such as aortic coarctation or left ventricular outflow tract obstruction [53].

Right-sided accessory pathways, classically associated with Ebstein's anomaly, have been reported in 25% and may be multiple [52,54,55].  In addition to AV reciprocating tachycardia, ectopic atrial tachycardia and atrial fibrillation or flutter can occur. Tachyarrhythmias may be poorly tolerated in patients with severe Ebstein's malformation and/or an associated ASDs that shunt right-to-left during tachycardia [5]. High risk or multiple pathways may support rapid conduction during atrial fibrillation or flutter [55]. Mapping and ablation can be challenging as signals in the atrialized portion of the right ventricle may be complex and the true AV groove, along which accessory pathways are targeted, may not be readily apparent. To identify the AV groove, coronary angiography may be performed or a thin multielectrode catheter may be inserted in the right coronary artery [56]. Of 65 patients with Ebstein's anomaly and accessory pathways, short-term ablation success rates ranged from 75% to 89%, depending on pathway location, with late recurrences in up to 32% [57].

### Univentricular hearts with Fontan palliation

The Fontan procedure has undergone multiple modifications to become the treatment of choice for various forms of single ventricle physiology  [25]. Sudden cardiac death of presumed arrhythmic etiology is a major cause of late mortality  [58]. Atrial arrhythmias are a challenge to manage, often incur substantial morbidity, and may be poorly tolerated hemodynamically. Although some respond favorably to pharmacological therapy  [10] results are often disappointing.

**Figure 2:**
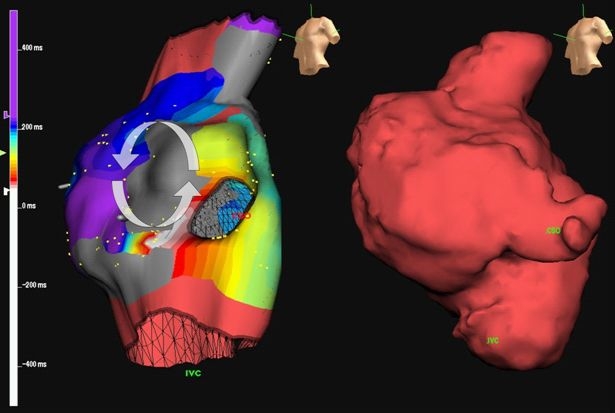
Electroanatomic mapping in a right atrium to pulmonary artery Fontan. An electroanatomic map (left) and imported CMR image (right) are shown in a patient with a classic modified Fontan and recalcitrant atrial tachyarrhythmias.  The grey regions denote areas of dense scar.  Local activation times are color-coded, from white to red, orange, yellow, green, light blue, dark blue, and purple.  Note the narrow channel of tissue between two dense scars.  The arrhythmia circuit propagated counterclockwise around the upper scar and was successfully interrupted by ablating this narrow isthmus. Reproduced from Khairy P. EP challenges in adult congenital heart disease Heart Rhythm 2008;5(10):1464-72. Copyright (2008), with  permission from Elsevier.

In general, the incidence of atrial tachyarrhythmias appears lower in patients with total cavo-pulmonary connections in comparison to classic right atrium to pulmonary artery connections. Studies comparing the relative incidence of atrial tachyarrhythmias in patients with lateral tunnel versus extracardiac conduits are ongoing. Overall, the most common arrhythmia is atrial macroreentry [59]. Although atrial fibrillation may occur, it is surprisingly less common than one would anticipate, especially considering the often extremely dilated right atrium. Tachycardia circuits may be complex and/or multiple [28,60,61]. Single circuits that are quite amenable to catheter ablation may occasionally be encountered (Figure 2). Overall acute success rates exceed 80% [62-64], although recurrences or new onset arrhythmias remain problematic, in the order of 30% to 45% within the first year [62,65].

**Figure 3:**
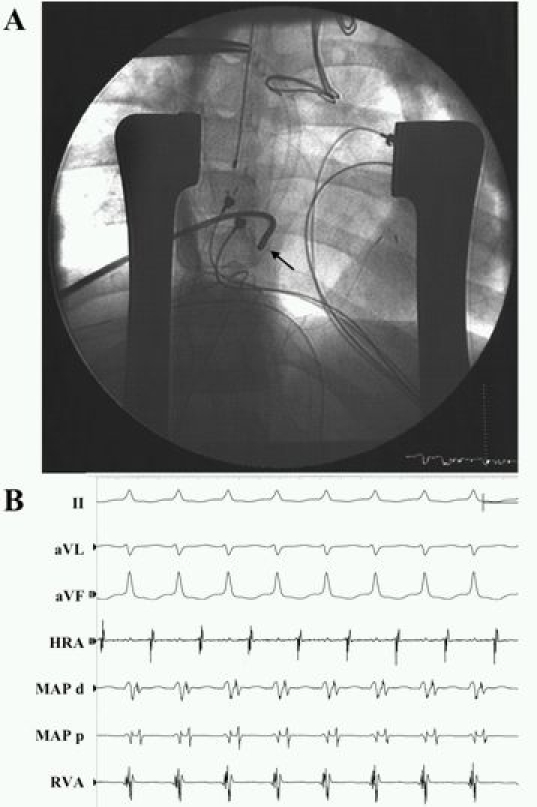
Catheter ablation in an extracardiac Fontan via a direct atriotomy approach. A patient with hypoplastic left heart syndrome and Ebstein's malformation of the right-sided  AV valve had poorly tolerated incessant orthodromic AV reciprocating tachycardia postoperatively after an extracardiac Fontan with surgical accessory pathway ligation.  In Panel A, an anteroposterior view is shown at the site of successful ablation, portraying the position of the radiofrequency ablation catheter (black arrow).  The sternum is splayed open by means of thoracic retractors.  Epicardial bipolar atrial and ventricular pacing leads are seen.  Shown in Panel B are recordings from surface ECG leads II, aVL, and aVF; epicardial high right atrium (HRA); distal (MAP d) and proximal (MAP p) electrode pairs of the radiofrequency ablation catheter; and epicardial ventricle (RVA).  Orthodromic AV reciprocating tachycardia is seen with the mapping catheter positioned at the site of successful ablation. Reproduced from Khairy P et al. Transcatheter ablation via a sternotomy approach as a hybrid procedure in a univentricular heart. PACE 2008;31(5):639-40, with permission from Wiley-Blackwell.

Patients with failing Fontans and refractory atrial arrhythmias should be considered for surgical conversion to a total cavopulmonary connection with concomitant arrhythmia surgery. This typically includes debulking the right atrium, removing thrombus, excising right atrial scar tissue, epicardial pacemaker implantation, a modified right atrial Maze procedure and, in patients with prior documented atrial fibrillation, a left-sided Maze procedure as well [66]. Case series with short-term follow-up report promising results, with arrhythmia recurrence rates of 13% to 30% [66-68]. Although the incidence of atrial tachyarrhythmias may be lower, extracardiac Fontans substantially complicate transvenous access to arrhythmia circuits, as exemplified by Figure 3 [69].

### Tetralogy of Fallot

The frequency and nature of arrhythmias encountered in tetralogy of Fallot are related, in part, to the type of surgical correction. Repairs were initially performed by means of a ventricular incision. This approach was largely abandoned in favor of transatrial/transpulmonary access to reduce risk for potentially fatal ventricular tachyarrhythmias [70]. The most common atrial circuit is typical clockwise or counterclockwise atrial flutter utilizing the sub-Eustachian isthmus between the tricuspid valve annulus and inferior vena cava, even if the P-wave morphology is not characteristic for these arrhythmias [71]. Other circuits often involve the lateral wall and may be multiple, often with a double-loop type of reentry. Non-automatic focal atrial tachycarrhythmias most commonly arise adjacent to suture points, with radial spread of activation. A practical approach to catheter ablation in tetralogy of Fallot has been previously described [71].

**Figure 4:**
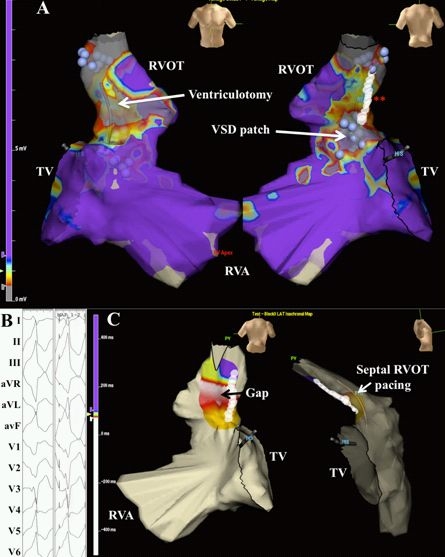
Voltage and pace mapping of ventricular tachycardia in tetralogy of Fallot. In Panel A, a color-coded voltage map of the right ventricle in sinus rhythm is shown in a patient with tetralogy of Fallot and ventricular tachycardia.  Values below 0.5 mV are depicted in grey and above 1.5 mV in purple.  The presumed ventriculotomy incision and ventricular septal defect patch are indicated by the white arrows.  In areas of low voltage, unipolar pacing at 10 mAmp/2 ms was performed.  Unexcitable tissue was marked by blue spheres.  Linear ablation (white circles at the double red asterisk) was performed connecting two unexcitable areas, i.e. pulmonary annulus and ventricular septal defect patch.  TV denotes tricuspid valve; RVA, right ventricular apex; RVOT, right ventricular outflow tract.  This site was selected for ablation following pace mapping of the septal RVOT, shown in Panel B.  Bouts of non-sustained ventricular tachycardia were inducible, corresponding to documented sustained events.  Left and right hand panels capture non-sustained ventricular tachycardia and successful 12-lead pace mapping, respectively.  In Panel C, block was verified by pacing from a decapolar catheter septal to the ablation line and mapping local activation.  Local activation times are color-coded from white to red, orange, yellow, green, light blue, dark blue, and purple.  Early activation along the mid-portion of the RVOT adjacent to the ablation line suggests a conduction gap.  Additional ablation was performed along the mid-portion of the line, until bidirectional block was achieved. Reproduced from Khairy P, Stevenson WG.  Catheter ablation in tetralogy of Fallot. Heart Rhythm 2009;6(7):1069-74.71. Copyright (2009), with  permission from Elsevier.

**Figure 5:**
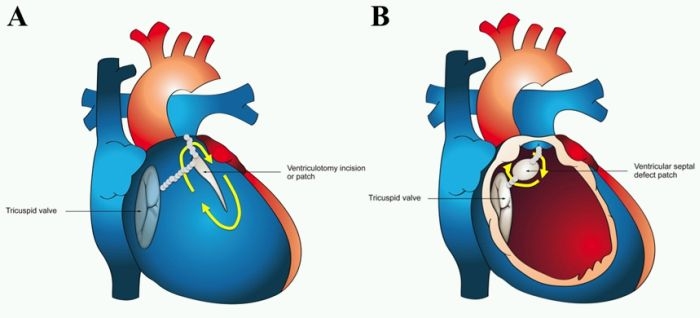
Potential critical isthmuses for ventricular tachycardia in surgically repaired tetralogy of Fallot. Potential arrhythmia circuits along the right ventricular free wall (Panel A) and septum (Panel B) are indicated by yellow arrows.  Small grey circles schematically represent four critical isthmuses for ventricular tachycardia that may be transected by catheter ablation. Reproduced from Khairy P, Stevenson WG.  Catheter ablation in tetralogy of Fallot. Heart Rhythm 2009;6(7):1069-74.71. Copyright (2009), with  permission from Elsevier.

**Figure 6:**
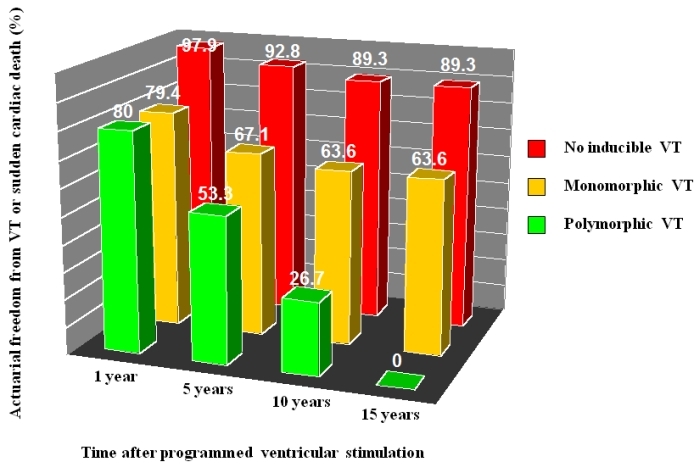
Value of programmed ventricular stimulation in tetralogy of Fallot. Actuarial freedom from ventricular tachycardia (VT) and sudden cardiac death is depicted in patients with no inducible VT, inducible monomorphic VT, and inducible polymorphic VT at 1, 5, 10, and 15 years following programmed ventricular stimulation. Adapted with permission from Khairy P et al. Value of programmed ventricular stimulation after tetralogy of fallot repair: a multicenter study. Circulation 2004;109(16):1994-2000.

Sudden cardiac death is the most common cause of mortality late after repair [72,73]. Monomorphic ventricular tachycardia occurs in approximately 10% of patients by 20 years of follow-up, an example of which is depicted in Figure 4  [71]. Nearly all forms are critically dependent on at least one of four discrete narrow channels, or isthmuses, portrayed in Figure 5  [71,74]. Considerable efforts have been directed towards identifying risk factors for ventricular tachycardia and sudden death. In the largest cohort study with a mean follow-up > 20 years, non-invasive risk factors were a QRS interval ≥ 180 ms, an annual increase in QRS duration, older age at repair, and presence of a right ventricular outflow tract patch [75]. Patients with ventricular tachycardia or sudden cardiac death were more likely to have increased cardiothoracic ratios, at least moderate pulmonary and tricuspid regurgitation, and peripheral pulmonary stenosis. A higher QT dispersion was also noted, believed to reflect increased heterogeneity in myocardial repolarization. Other reported risk factors include frequent ectopic beats [76] increased right ventricular systolic pressures [77-79] complete heart block [77,80] and increased JT dispersion [81,82]. In patients deemed at moderate risk, further risk stratification by means of programmed ventricular stimulation may be helpful [83-85]. Event-free survival rates in non-inducible and inducible patients are shown in Figure 6  [83].

**Figure 7:**
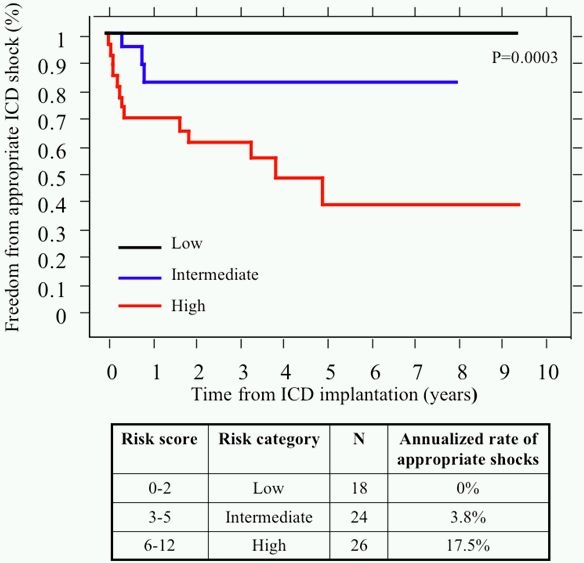
Freedom from appropriate ICD shocks in primary prevention patients with tetralogy of Fallot according to their risk category. In patients with primary prevention indications, Kaplan-Meier survival curves for freedom from first appropriate ICD shock are plotted and compared according to risk score classification.  Risk score, corresponding risk category, number of patients, and annualized rate of appropriate shocks are summarized below. Reproduced with permission from Khairy P et al. Circulation 2008;117(3):363-70.

Implantable cardioverter-defibrillators (ICD) are increasingly utilized in the primary and secondary prevention of sudden death in patients with tetralogy of Fallot, with patients experiencing relatively high rates of appropriate shocks  [86]. For patients with primary prevention indications, a risk score was derived from surgical, hemodynamic, electrocardiographic, and electrophysiological factors (Table 1)  [86]. As depicted in Figure 7, patients with < 3 points (low risk) experienced no appropriate shocks. In patients with 3-5 points (intermediate risk) and > 5 points (high risk), appropriate shocks were received by 3.8% and 17.5% of patients per year, respectively  [86].

### D-transposition of the great arteries (D-TGA)

**Figure 8:**
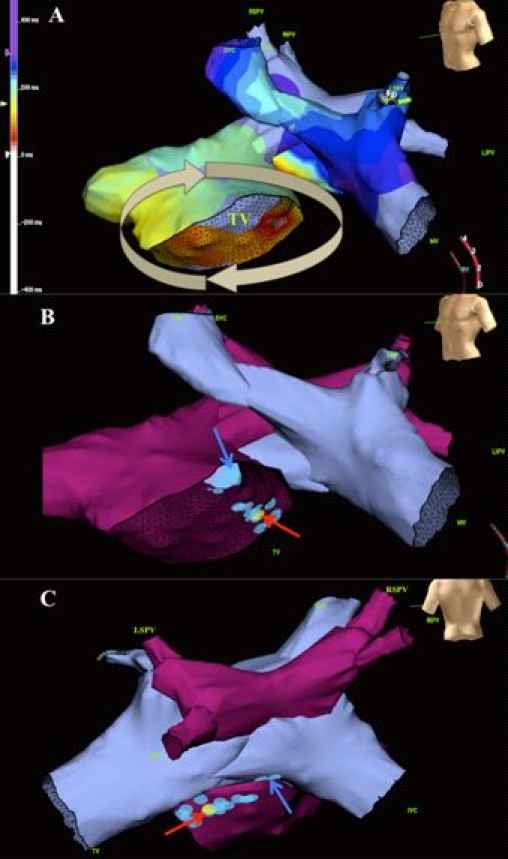
Ablation of the cavotricuspid isthmus in D-TGA with Mustard baffle. Panel A depicts an electroanatomic map of systemic and pulmonary venous atria.  Local activation times are color-coded, from white to red, orange, yellow, green, light blue, dark blue, and purple.  A circuit rotates clockwise around the tricuspid valve (TV).  The anatomical relationship between systemic (blue) and pulmonary (purple) venous atria is demonstrated in left anterior oblique (Panel B) and posterior (Panel C) views.  Blue circles mark ablation sites and the yellow circle the site of successful arrhythmia termination.  Blue and red arrows indicate ablation lesions within systemic and pulmonary venous portions of the cavotricuspid isthmus, respectively. Reproduced from Khairy P, Van Hare G.  Catheter ablation in transposition of the great arteries with Mustard or Senning baffles. Heart Rhythm 2009;6(2):283-9. Copyright (2009), with  permission from Elsevier.

Although arterial switch surgery has supplanted atrial redirection as the procedure of choice for D-TGA, most adults with D-TGA have had intraatrial baffle repairs of the Mustard or Senning variety. Sinus node dysfunction is highly prevalent with increasing age, with loss of sinus rhythm in 60% at 20 years  [87]. In a meta-analysis comparing outcomes in 885 patients from 7 studies, sinus node dysfunction was more common in patients with Mustard procedures  [88]. Atrial tachyarrhythmias have been reported in 24% of patients at 20 years  [87] with similar rates in patients with Mustard and Senning baflles  [88]. Sudden death is the leading cause of late mortality after intraatrial baffle surgery  [6,87,89,90]. Recent reports suggest that atrial tachyarrhythmias are an important contributor to sudden death  [91]. Contributing factors may include longer cycle lengths than typical atrial flutter (favoring 1:1 conduction), impaired AV transport with failure to augment right ventricular filling rates during tachycardia  [92], systemic right ventricular dysfunction  [93] and subendocarial ischemia resulting from a right coronary circulation irrigating a systemic ventricle  [94]. As exemplified by Figure 8, catheter ablation of atrial arrhythmias frequently requires access to the pulmonary venous atrium and is often successful  [95].

Studies are beginning to address the role of ICDs  [91] and cardiac resynchronization therapy for failing systemic right ventricles  [16,96]. Unfortunately, risk stratification for sudden death remains ineffective. In a retrospective multicenter case-control study, risk factors were limited to the presence of arrhythmia or heart failure symptoms and history of documented atrial fibrillation or flutter  [97]. The electrocardiogram, chest x-ray, and Holter findings were not predictive of sudden death, and medical therapy and pacemakers were not found to be protective. Moreover, in a multicenter cohort study, very few appropriate ICD shocks were received in patients with primary prevention indications  [91]. The presence or degree of right ventricular dysfunction was not associated with appropriate shocks. Encouragingly, beta-blocker therapy appeared protective.

## Summary

Patients with congenital heart disease represent a heterogeneous population with varied arrhythmic diagnoses and issues. The last decade has witnessed major advances in our understanding of arrhythmia mechanisms and therapeutic options. Sudden cardiac death of presumed arrhythmic etiology is the leading cause of mortality, particularly in patients with left-sided obstructive lesions, D-TGA and intraatrial baffles, tetralogy of Fallot, and severe systemic ventricular dysfunction. Sinus node dysfunction is common in left atrial isomerism and with Mustard, Senning, Glenn, or Fontan surgery. Complete AV block frequently occurs in patients with L-looped ventricles, left atrial isomerism, and AVCD. Atriotomy incisions, sutures, baffles, and conduits may predispose to the development of scar-based macroreentrant atrial circuits, as commonly encountered in surgically repaired ASD, Mustard and Senning baffles, tetralogy of Fallot, and Fontan palliation.

ICDs and cardiac resynchronization therapy are increasingly utilized in patients with congenital heart disease. Unlike standard indications for patients without congenital heart disease, evidence supporting this technology is limited but growing. Guidelines have begun incorporating issues relevant to this patient population and targeted training programs are now offered. A thorough understanding of conduction system variants, arrhythmia mechanisms, underlying anatomy, and physiology is important to safely and effectively manage arrhythmias in this unique and diverse patient population.

## Figures and Tables

**Table 1:**
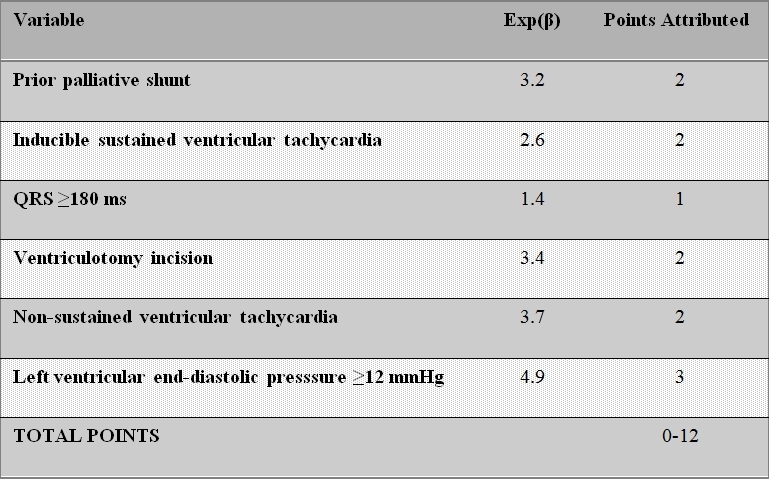
Risk score for appropriate ICD shocks in patients with tetralogy of Fallot and primary prevention indications
